# The role, mechanism and potentially novel biomarker of microRNA-17-92 cluster in macrosomia

**DOI:** 10.1038/srep17212

**Published:** 2015-11-24

**Authors:** Jing Li, Liping Chen, Qiuqin Tang, Wei Wu, Hao Gu, Lou Liu, Jie Wu, Hua Jiang, Hongjuan Ding, Yankai Xia, Daozhen Chen, Yali Hu, Xinru Wang

**Affiliations:** 1 State Key Laboratory of Reproductive Medicine, Institute of Toxicology, Nanjing Medical University, Nanjing 211166, China; 2 Key Laboratory of Modern Toxicology of Ministry of Education, School of Public Health, Nanjing Medical University, Nanjing 211166, China; 3 Department of Public Health, Xuzhou Medical College, Xuzhou, Jiangsu, China; 4 Department of Gynecology and Obstetrics, The Second Affiliated Hospital of Nantong University, Nantong, 226001, China; 5 Reproductive Medicine Center, The Affiliated Drum Tower Hospital of Nanjing University Medical School, Nanjing, 210008, China; 6 State Key Laboratory of Reproductive Medicine, Department of Obstetrics, Nanjing Maternity and Child Health Care Hospital Affiliated to Nanjing Medical University, Nanjing 210004, China; 7 State Key Laboratory of Reproductive Medicine, Wuxi Maternal and Child Health Care Hospital Affiliated to Nanjing Medical University, Wuxi 214002, China; 8 Perinatology Unit, Changzhou Maternity and Child Health Care Hospital Affiliated to Nanjing Medical University, Changzhou 213003, China

## Abstract

Macrosomia is one of the most common perinatal complications of pregnancy and has life-long health implications for the infant. microRNAs (miRNAs) have been identified to regulate placental development, yet the role of miRNAs in macrosomia remains poorly understood. Here we investigated the role of miR-17-92 cluster in macrosomia. The expression levels of five miRNAs in miR-17-92 cluster were significantly elevated in placentas of macrosomia, which may due to the up-regulation of miRNA-processing enzyme *Drosha* and *Dicer*. Cell cycle pathway was identified to be the most relevant pathways regulated by miR-17-92 cluster miRNAs. Importantly, miR-17-92 cluster increased proliferation, attenuated cell apoptosis and accelerated cells entering S phase by targeting SMAD4 and RB1 in HTR8/SVneo cells. Furthermore, we found that expression of miR-17-92 cluster in serum had a high diagnostic sensitivity and specificity for macrosomia (AUC: 80.53%; sensitivity: 82.61%; specificity: 69.57%). Our results suggested that miR-17-92 cluster contribute to macrosomia development by targeting regulators of cell cycle pathway. Our findings not only provide a novel insight into the molecular mechanisms of macrosomia, but also the clinical value of miR-17-92 cluster as a predictive biomarker for macrosomia.

Macrosomia is one of the most common pleiotropic complications of pregnancy. It is characterized by the birth weight over 4,000 g or above the 90th percentile for gestation^1^. The frequency of macrosomia has been reported to increase rapidly in China^2^. Fetal macrosomia was not only associated with prolonged labour, cesarean section, post-partum infection, and postpartum haemorrhage for mothers, but also with higher risk of birth asphyxia, shoulder dystocia, neonatal hypoglycemia, clavicular fracture, and perinatal mortality for infants^3^,^4^. In addition, macrosomic fetuses may have an increased susceptibility to childhood obesity, diabetes and cardiovascular diseases during adulthood^5^,^6^.

Multiple factors have been identified that can affect fetal birth weight, such as genetic factors, various growth factors, hormones, nutrition and placental factors^7^. As an important interface between the fetal and maternal environments, placenta mediates the efficient maternal-to-fetal transfer of gases, nutrients and waste products. The development of placenta depends on proper regulation of trophoblast proliferation, differentiation and invasion^8^. Studies have found that fetal growth is closely related to placental development^9^. In diabetic pregnancy, placental weight is higher compared with normal pregnancy^10^. Numbers of syncytiotrophoblast nuclei, syncytial knots, and fibrinoid necrosis were also increased in placentas of macrosomia^11^. Dysregulation of trophoblast proliferation may contribute to abnormal growth of placenta^12^. However, the molecular mechanisms of its regulation are largely unknown.

microRNAs (miRNAs) are small endogenous noncoding RNAs, processed through a series of sequential steps involving the enzymes Drosha and Dicer. miRNAs act as post-transcriptional regulators of gene expression through translation inhibiting or degradation by base-pairing to the 3′-untranslated region^13^. Accumulating evidence suggested that miRNAs participate in various biological mechanisms by negatively regulating their mRNA targets^13^,^14^. It has been shown that miRNAs are abundantly expressed in placenta, and contribute to regulating placental development^15^. Dysregulated miRNAs expression in placenta have been associated with a variety of pregnancy complications, such as preeclampsia (PE)^16^, fetal growth restriction^17^, preterm birth^18^ and macrosomia^19^.

Human miR-17-92 cluster located at 13q31.3, comprising six miRNAs: miR-17, miR-18a, miR-19a, miR-19b, miR-20a and miR-92a. Previous study has shown that miRNAs in miR-17-92 cluster plays play an important role in cell cycle progression, proliferation, apoptosis, invasion and migration^20^. Meanwhile, dysregulation of miRNA-17-92 cluster in placentas might also be responsible for various pregnancy diseases (e.g. PE^21^, early pregnancy loss^22^). In the present study, we explored the expression of miR-17-92 cluster in placentas and maternal serum of macrosomia and normal controls. Furthermore, the role of miR-17-92 cluster in proliferation, apoptosis and cell cycle were also investigated in HTR8/SVneo cells. Our results suggest that expression levels of miR-18a, miR-19a, miR-20a, miR-19b and miR-92a were significantly higher in placentas of macrosomia, and elevated expression of miR-17-92 increased HTR8/SVneo cells proliferation, attenuated cell apoptosis and accelerated cells entering S phase. miR-17-92 cluster miRNAs contribute to macrosomia development by targeting regulators of cell cycle pathway. Notably, miR-17-92 cluster may be a potential biomarker for the diagnosis of macrosomia.

## Results

### Clinical data

Study population characteristics are described in Additional file 1. The birth weight was significantly higher in neonates with macrosomia than that in normal controls, while pre-pregnant body mass index was significantly lower in group of macrosomia. No significant difference was observed between neonates with macrosomia and normal controls with regard to maternal age, gestation weeks, weight gain during pregnancy and infant gender distribution.

### Expression of miR-17-92 cluster in placentas

**Figure 1:**
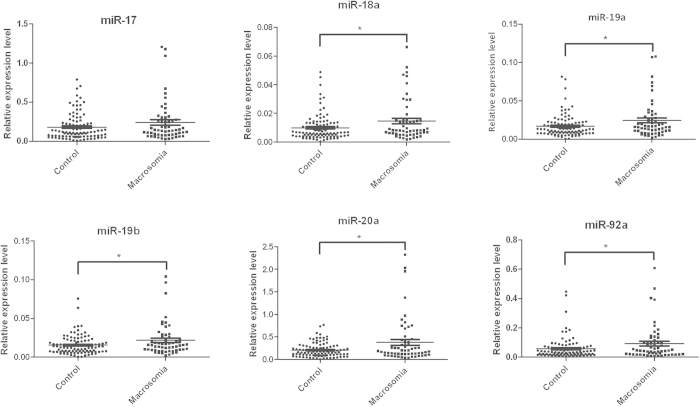
miR-17-92 cluster miRNAs were up-regulated in placenta tissues of macrosomia. Expression levels of miR-17, miR-18a, miR-19a, miR-19b, miR-20a and miR-92a were analyzed by qRT-PCR in placentas of macrosomia infants (n = 57) and controls (n = 100), and normalized to RNU6B. *P < 0.05; compared with the controls.

The expression levels of miR-17-92 cluster miRNAs in placentas of 100 normal controls and 57 neonates with macrosomia were examined using quantitative RT-PCR (qRT-PCR). As shown in Fig. 1, miR-18a, miR-19a, miR-20a, miR-19b and miR-92a were significantly increased in neonates with macrosomia compared with normal controls (Fig. 1). However, no significant difference was found in the expression level of miR-17 between two groups.

### Identification of the target genes and pathways of miR-17-92 cluster

**Figure 2:**
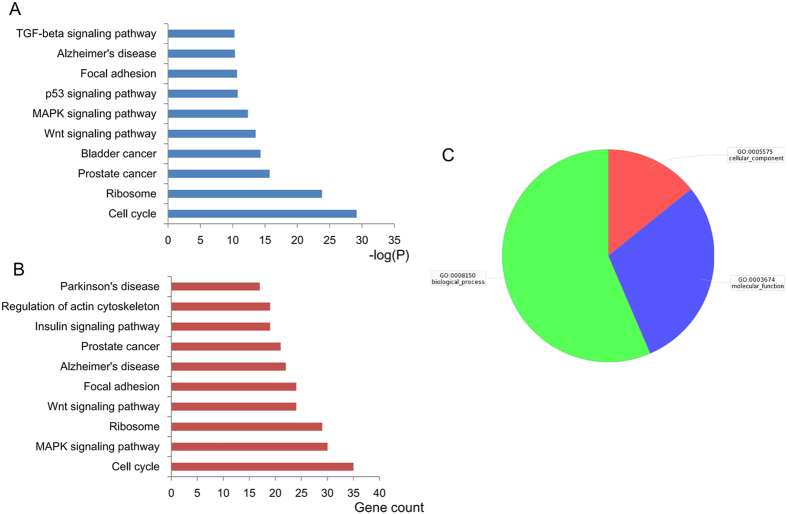
Target genes and pathways of the related miR-17-92 cluster miRNAs in macrosomia were enriched. Top ten KEGG pathways regulated by miR-17-92 cluster are enriched according to *P* value (**A**) or gene count (**B**). (**C**) GO terms are grouped into three categories.

**Figure 3:**
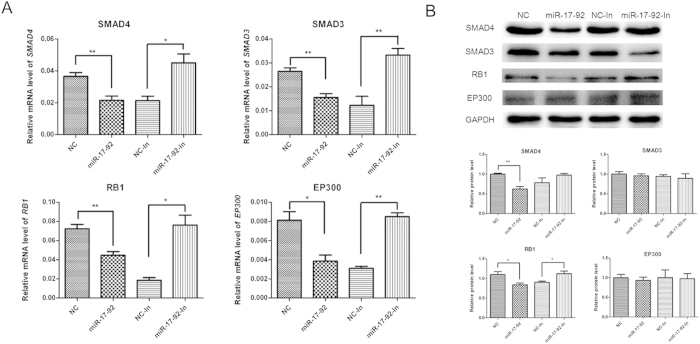
miR-17-92 cluster miRNAs inhibited expression of SMAD4 and RB1 in HTR8/SVneo cells. (**A**) Detection of *SMAD4, SMAD3, RB1*, and *EP300* mRNA expression in miR-17-92 mimics or miR-17-92 inhibitors-transfected HTR8/SVneo cells by qRT-PCR, and normalized to *GAPDH*. (**B**) Detection of SMAD4, SMAD3, RB1, and EP300 protein expression by Western-blot. Data were normalized to the level of GAPDH. *P < 0.05; **P < 0.01; compared with the control.

To further understand the potential role of the cluster miRNAs (miR-18a, miR-19a, miR-20a, miR-19b and miR-92a), we performed the enrichment analysis using the Gene Ontology (GO) and KEGG pathway analysis. GO terms were grouped into three categories: biological process, molecular function and cellular component. Enriched common biological pathways included cell cycle, ribosome, prostate cancer, bladder cancer, Wnt signaling pathway, and so on. Of note, cell cycle pathway included the largest numbers of targets gene and the smallest *P* value (Fig. 2). Detailed information of miR-17-92 target genes in cell cycle pathway were listed in Additional file 2. Increased expression levels of miR-17-92 cluster miRNAs were confirmed by qRT-PCR when transfected into HTR8/SVneo cells. The introduction of miR-17-92 mimics dramatically increased the expression of endogenous miR-18a, miR-19a, miR-19b, miR-20a and miR-92a in HTR8/SVneo cells, and the inhibitors significantly decreased the expression of these miRNAs (Additional file 3). To identify the potential target genes of miR-17-92 cluster, we analyzed the mRNA expression of all the 35 target genes in cell cycle pathway. Six genes (*SMAD4*, *SMAD3*, *CDKN1A*, *RB1*, *MAD1L1* and *EP300*) were significantly down-regulated in miR-17-92 mimics-transfected cells and up-regulated in miR-17-92 inhibitors-transfected cells (Additional file 4). Since miRNAs function by suppressing the expression of their target genes, these critical cell cycle regulators were further examined in placentas. Expression levels of *SMAD4*, *SMAD3*, *RB1* and *EP300* genes were significantly decreased in placentas of neonates with macrosomia compared with those of controls (Additional file 5). Furthermore, Western-blot analysis revealed that protein expression levels of SMAD4 and RB1 were appreciably lower in miR-17-92 mimics-transfected HTR8/SVneo cells and higher in miR-17-92 inhibitors-transfected cells. However, SMAD3 and EP300 levels did not shown any significant difference (Fig. 3). In addition, protein expression levels of SMAD4 and RB1 in macrosomia placentas were also lower than that in controls, indicating that SMAD4 and RB1 may play important roles in macrosomia (Additional file 5).

### Targeting SMAD4 and RB1 by miR-17-92 cluster in HTR8/SVneo cells

**Figure 4:**
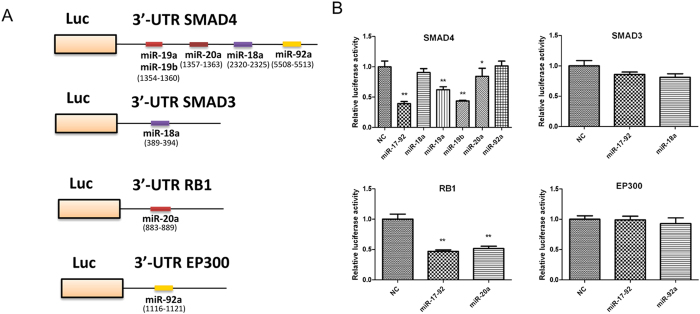
miR-17-92 cluster miRNAs targeted SMAD4 and RB1 in HTR8/SVneo cells. (**A**) A schematic presentation of putative miR-17-92 cluster miRNAs-binding sites in the 3′-UTR regions of SMAD4, SMAD3, RB1 and EP300. (**B**) Luciferase assay in which the activities of 3′-UTRs of SMAD4, SMAD3, RB1 and EP300 fused to the luciferase gene constructs were measured in the presence of individually miR-17-92 cluster miRNAs or miR-17-92 cluster mix in HEK 293T cells. Data are expressed as the mean ± SEM (n = 3). *P < 0.05; **P < 0.01; compared with the control.

To determine how miR-17-92 regulates SMAD4 and RB1 expression, we employed luciferase reporter assays in which the activity of the luciferase gene fused to 3′-UTR of SMAD4, SMAD3, RB1 and EP300 (Fig. 4). As shown in Fig. 4B, luciferase reporter activities obviously decreased when HEK 293 T cells were cotransfected with the miR-17-92 cluster mix, miR-19a, miR-19b and miR-20a and pGL3-SMAD4, and also attenuated when HEK 293 T cells were cotransfected with the miR-17-92 cluster mix, miR-20a and RB1 construct (Fig. 4).

Unexpectedly, despite the bioinformatics prediction, there were no significant changes in the expression of luciferase reporter activities in HEK 293 T cells cotransfected with the miR-17-92 cluster and pGL3-SMAD3 or pGL3-EP300 (Fig. 4). These data implied that miR-19a and miR-19b may regulate SMAD4 protein together, while miR-20a may regulate SMAD4 and RB1 protein by directly binding to the 3′-UTR region of these target genes.

### Functional effects of miR-17-92 cluster on proliferation, apoptosis and cell cycle in HTR8/SVneo cells

**Figure 5:**
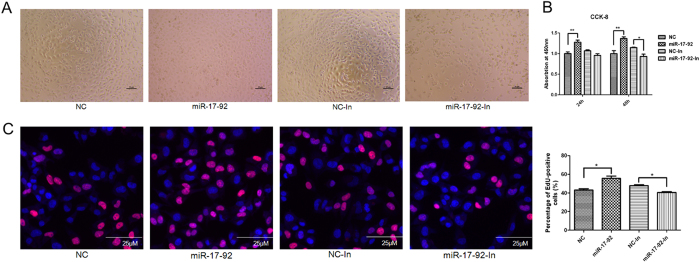
miR-17-92 cluster miRNAs enhanced the proliferation of HTR8/SVneo cells. The effect of miR-17-92 cluster miRNAs on cell proliferation was detected using CCK-8 and EdU after transfection with miR-17-92 mimics or inhibitors. (**A**) Phase contrast microscopy of HTR8/SVneo cells treated with miR-17-92 mimics or inhibitors for 24h. (**B**) The effect of miR-17-92 cluster miRNAs on cell proliferation was detected at 24h and 48h using CCK-8. (**C**) Cell proliferation was determined by the EdU assay. Forty-eight hours after transfection, HTR8/SVneo cells were stained with EdU and DAPI. The percentage of EdU-positive HTR8/SVneo cells was quantified. Data are expressed as the mean ± SEM (n = 5). *P < 0.05; **P < 0.01; compared with the control.

CCK-8 and EdU assays were performed to estimate the effects of miR-17-92 cluster on HTR8/SVneo cells proliferation. CCK-8 and EdU assays both showed that the proliferation was significantly increased in HTR8/SVneo cells transfected with miR-17-92 mimics compared to controls, whereas decreased in inhibitors at 48 h (Fig. 5).

**Figure 6:**
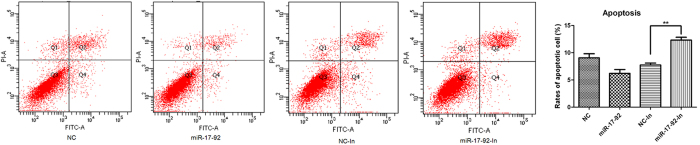
miR-17-92 cluster miRNAs attenuated apoptosis of HTR8/SVneo cells. Apoptosis was detected by flow cytometry with Annexin V-FITC staining in miR-17-92 mimics or miR-17-92 inhibitors-transfected HTR8/SVneo cells for 48h. Percentage of apoptotic cells were analyzed by FACS Calibur Flow Cytometer. Data are expressed as the mean ± SEM (n = 3). **P < 0.01; compared with the control.

Next we investigated whether miR-17-92 had an effect on apoptosis of HTR8/SVneo cells. Inhibition of miR-17-92 significantly increased the apoptosis compared with the negative control (Fig. 6), while up-regulation of miR-17-92 inhibited this process, though not statistically significant.

**Figure 7:**
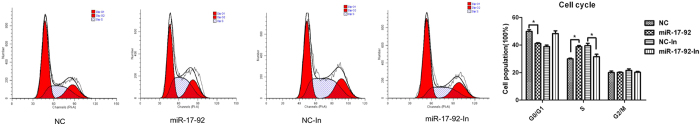
miR-17-92 cluster miRNAs affected cell cycle in HTR8/SVneo cells. The cell cycle was detected by flow cytometry with PI staining in HTR8/SVneo cells after transfection with miR-17-92 mimics or inhibitors for 48 h. The percentage of cells in G1, S, and G2 cell cycle phases were depicted in the bar graph. Data are expressed as the mean ± SEM (n = 3). *P < 0.05; compared with the control.

Furthermore, cell cycle analysis showed that ectopic expressions of miR-17-92 cluster miRNAs decreased the proportion of HTR8/SVneo cells in the G0/G1. More cells entered S phase after introducing miR-17-92 cluster miRNAs. Meanwhile, inhibitors caused the arrest of cells in G0/G1 phase (Fig. 7), suggesting that miR-17-92 cluster was involved in the regulation of cell cycle progression in HTR8/SVneo cells.

### Expression of *Drosha* and *Dicer* in placentas

Since *Drosha* and *Dicer* are essential for the processing of miRNAs, we proposed whether *Drosha* and *Dicer* were responsible for the aberrant expression of miR-17-92 cluster miRNAs in macrosomia. We examined the expression levels of *Drosha and Dicer* in placentas of the two groups. The result showed that their expression levels were significantly elevated in placentas of neonates with macrosomia compared with normal controls (Additional file 6).

### Expression of serum miR-17-92 cluster and its potential diagnosis value for macrosomia

**Figure 8:**
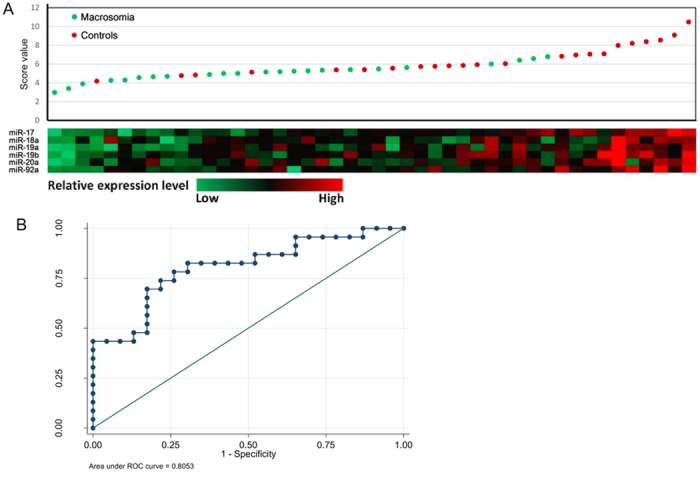
Risk-score and ROC curves for the ability of the maternal serum expression of six miRNAs in miR-17-92 cluster to differentiate the macrosomia from the controls. (**A**) Risk-score distribution and color-gram of serum-miRNA expression profiles of macrosomia were performed. Rows represent miRNAs and columns represent subjects. Green denotes down-regulated expression and red denotes up-regulated expression compared with the mean. (**B**) ROC curve was shown for the ability of the six miRNAs to differentiate macrosomia from the controls.

**Figure 9:**
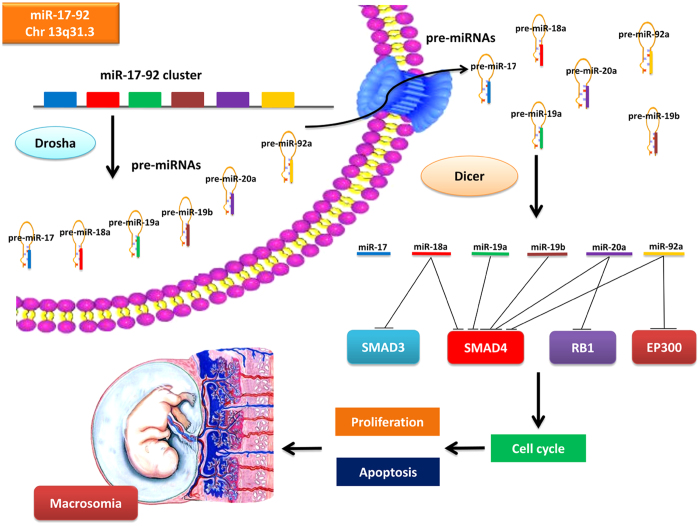
A model was presented for miR-17-92 cluster mediated macrosomia. The miR-17-92 cluster encodes for six distinct miRNAs. Drosha executes the initial step of miRNA processing in the nucleus, and the resultant pre-miRNAs are exported to the cytoplasm where they are cleaved by Dicer to generate the final mature miRNAs. Up-regulation of *Drosha* and *Dicer* causes miR-17-92 overexpression, which drives macrosomia by inhibiting multiple negative regulators of cell cycle pathway that mediated the pathology of macromosia.

We found that all the six miRNAs of miR-17-92 cluster were expressed in maternal serum. The expression levels of miR-17, miR-18a, miR-19a and miR-92a were significantly lower in neonates with macrosomia than in normal controls (Additional file 7). To evaluate the diagnostic value of the miR-17-92 cluster miRNAs in maternal serum, we performed a risk scoring procedure on their data sets. The distribution of miR-17-92 cluster miRNAs expression levels and risk scores of the all combined subjects are shown in Fig. 8. It can be noted that macrosomia tended to have lower expression levels of the miR-17-92 cluster miRNAs (Fig. 8). By using the 95% reference interval of each miRNA expression value as a risk score, we constructed the receiver operating characteristic (ROC) curves and calculated the Area Under the ROC Curve (AUC) to assess the sensitivity and specificity for prediction. As depicted in Fig. 8, when the six miR-17-92 cluster miRNAs were utilized to separate the control and macrosomia groups, the AUC was 80.53% (sensitivity: 82.61%; specificity: 69.57%) (Fig. 8). The overall model of miR-17-92 cluster miRNAs mediated macrosomia is shown in Fig. 9.

## Discussion

Recent studies have suggested that many miRNAs regulate trophoblast cell proliferation and apoptosis during placental development, and their abnormal expressions are associated with defective placentation^23^,^24^. Mutation of the miR-17-92 cluster in mice resulted in smaller mouse embryos and postnatal death^25^. A previous study has identified that miR-17 and miR-19b were down regulated in early pregnancy loss^22^. Additionally, high expression levels of miRNA-17 family miRNAs have been reported in placentas complicated by PE compared with healthy pregnancies^26^. The down-regulation of miR-92a in gestational diabetes mellitus placentas was also identified by another group^27^. In the current study, we focused on miR-17-92 cluster to determine if they were similarly disrupted in placental dysregulation of macrosomia. The results showed that five miRNAs in miR-17-92 cluster (miR-18a, miR-19a, miR-19b, miR-20a and miR-92a) were significantly higher in placentas of macrosomia.

In the process of miRNA genesis, two members of the double-stranded RNA-specific endonuclease family, Drosha and Dicer, convert precursor forms of microRNAs into their mature forms using a stepwise process collaboratively. Our study indicated that *Drosha* and *Dicer* were up-regulated in pregnancies compromised by marosomia. Therefore, it is postulated that dysregulated miR-17-92 cluster may participate in the onset of the disease, and the up-regulated *Drosha* and *Dicer* enhanced the processing of miR-17-92 cluster genesis.

miR-17-92 cluster has been identified as an oncogenic RNA cluster, and its anti-apoptotic and proliferative effects were well investigated in many cancers^28^,^29^. Cytotrophoblasts share properties with tumor cells in rapid proliferation, invade maternal blood vessels and acquisition of a rich blood supply^30^. We postulated that aberrant proliferation of cytotrophoblasts may supply the excess nutrients that actually needed by the fetus exceedingly, leading to fetal macrosomia. Previous studies have reported that increased proliferation and decreased apoptosis were observed in villous part of diabetic placentas^12^. In addition, high expression levels of miR-17-92 cluster miRNAs were found to promote human cytotrophoblasts proliferation^31^. However, the role of miR-17-92 cluster in macrosomia remains poorly understood. In this study, exogenetic expression of miR-17-92 cluster increased proliferation, attenuation cell apoptosis, and enhanced cell cycle progression in HTR8/SVneo cells, which support the previous hypothesis.

The bioinformatics analysis predicted that miR-17-92 cluster may target cell cycle pathway, by which miRNAs control cell proliferation^32^. qRT-PCR and Western-blot showed a reduction in both SMAD4 and RB1 expression in the presence of miR-17-92 overexpression. Moreover, luciferase activity assay confirmed that miR-19a and miR-19b may regulate SMAD4 protein together, while miR-20a may regulate SMAD4 and RB1 protein directly.

SMAD4, the key components of TGF-β signaling transduction pathway, was found as the frequently mutated tumor suppressor genes in human tumors. Increased expression level of SMAD4 can induce apoptosis. Phosphorylated Smad2 and Smad3 oligomerize with Smad4 to form the Smad complex, and then translocate to the nucleus and binds nuclear DNA, leading to transcriptional activation of the target gene^33^. Although SMAD4 and SMAD3 have been consistently reported and predicted as the target genes of miR-17-92 cluster^34^,^35^, luciferase assay did not showed that miR-17-92 cluster regulated SMAD3 directly. Previous work has shown that SMAD4 was observed in normal villous trophoblast and choriocarcinoma^36^. In addition, another study has demonstrated the role of SMAD4 in the TGF-β1 pathway contributing to the invasiveness and metastasis of placental choriocarcinoma^37^. Therefore, regulation of SMAD4 by miR-19a, miR-19b and miR-20a may play an important role in the development of macrosomia.

RB1, a nuclear phosphoprotein, act as an inhibitor of cell proliferation, playing an important role in regulating apoptosis and cell cycle. The biological function of RB1 is mediated by interacting with transcription factors, p53 and MDM2^38^. Reduction of RB1 expression in the mouse resulted in excessive proliferation of trophoblast cells and a severe disruption of the normal labyrinth architecture in the placenta^39^. Furthermore, increased RB1 expression has been indicated to reduce trophoblast proliferation in fetal growth restriction^40^. We here demonstrated that miR-20a repressed the expression of RB1 directly. The suppression of RB1 may partly cause the excessive trophoblast proliferation in macrosomia.

Interactions between signaling pathways and miRNAs are complex. Besides cell cycle pathway, miR-17-92 cluster miRNAs have been found to regulate several other pathways, such as prostate cancer, bladder cancer, Wnt signaling pathway. The members of miR-17-92 cluster have been recognized to function in a cooperative and additive manner amongst others in the regulation of the target genes. Therefore, further studies about interactions between miRNAs and pathways involved in should be taken into consideration to clarify the miRNA regulating mechanisms of macrosomia.

Recently, it has been shown that circulating fetal nucleic acids in maternal serum and plasma might serve as biomarkers for noninvasive prenatal diagnosis^41^. miRNAs can be released into the circulation in apoptotic bodies or exosomes^42^. It would be useful to investigate whether the aberrant expression of miRNAs in placentas of macrosomia are also reflected in maternal serum or plasma. Thus, we examined the expression level of miR-17-92 cluster miRNAs in maternal serum, and found lower expression level of miR-17, miR-18a, miR-19a and miR-92a in the macrosomia pregnant women. To date, several studies examined the role of serum miRNAs in predicting macrosomia^43^,^44^,^45^. miR-17-92 cluster miRNAs (miR-20a, miR-19a, miR-19b) were found to be down-regulated in miRNA TaqMan Low Density Array analysis, but did not pass in the validation and discovery stage^44^. In addition, miR-18a-5p were confirmed to be down-regulated in plasma from pregnant women with macrosomia, and miR-17-92 cluster was also found to be significantly differentially expressed in macrosomia^45^. These findings indicate that miR-17-92 cluster might be a potential predictive tool for macrosomia. Although higher expression level of miR-17-92 cluster was found in placentas, for serum, the opposite trend was observed. It was also observed that miRNA level in tissue was not completely consistent with that in serum in some placental dysfunctions^44^,^46^.

Intensive studies have provided evidence that first trimester primary human trophoblasts are capable of secreting the exosome nanovesicles, which are known to contain miRNAs^47^,^48^. Thus, although we do not have mechanistic proof for this anticipation, it might be that less apoptosis in the placentas of macrosomia alters exosome-dependent or independent release of miRNAs into the maternal serum. Further mechanistic studies and large sample sizes are needed to prove this assumption. Given that the overall model of miR-17-92 cluster miRNAs could separate well of macrosomia and normal controls, we proposed that combination of miR-17-92 cluster expression may serve as a potential non-invasive biomarker for macrosomia diagnosis.

Our data suggest that aberrantly elevated expression of miR-17-92 cluster miRNAs may contribute to the pathogenesis of macrosomia and that the overall model of miR-17-92 cluster miRNAs in serum may have great potential for the detection and diagnosis of macrosomia. Together, our findings provide a foundation for interpretation of miRNA changes associated with macrosomia and extend the current concepts of macrosomia pathogenesis.

## Materials and Methods

### Human material samples and ethics

A total of 157 subjects, including 57 with non-diabetes macrosomia and 100 normal birth weight controls who undergoing cesarean section were included from Maternity and Child Health Care Hospitals Affiliated to Nanjing Medical University between January 2013 and December 2013. The diagnosis of macrosomia was defined by birth weight ≥4000 g. The mothers had no diabetes during pregnancy. The tissues were immediately frozen in liquid nitrogen following delivery, and then stored at −80 °C. About 50 mg tissues were kept in TRIzol for RNA extraction. The study was conducted according to the guidelines laid down in the Declaration of Helsinki and approved by the Ethics Committee of Nanjing Medical University. Written informed consent was obtained from all the pregnant women prior to participation.

### RNA extraction and quantitative RT- PCR

Total RNA, including miRNA, was extracted from placenta tissues, cell line and maternal serum using TRIzol Reagent (Invitrogen Life Technologies Co, CA, USA) according to manufacturer’s instructions. For miR-17-92 cluster detection, 500 ng of total RNA was reverse transcribed to cDNA using PrimeScript™ RT reagent Kit (Takara Bio, Tokyo, Japan), and RNU6B was analyzed as an internal control. For mRNA detection, reverse transcription was performed using PrimeScript™ RT Master Mix (Takara Bio, Tokyo, Japan), PCR analysis was then performed using SYBR^®^ *Premix Ex Taq*™ and normalized to the GAPDH according to the manufacturers guidelines. The sequences of primers for PCR are shown in Additional file 8 and Additional file 9.

### miRNA targets prediction and pathway enrichment analyses

In order to find out potential target genes of miR-17-92 cluster, we collected the target genes of the involved miRNA members from the miRTarBase database^49^, and then queried for Gene Ontology and KEGG pathway enrichment using CapitalBio^®^ Molecule Annotation System V3.0.

### Cell culture and transfection

The human placenta trophoblast cell line HTR-8/SVneo was cultured in RPMI 1640 media (Hyclone, UT, USA) with 10% fetal bovine serum at 37 °C in an atmosphere containing 5% CO_2_. To analyze the effect of miR-17-92 cluster on related genes expressions, cells were seeded in 24-well plates respectively and incubated overnight. When cells were grown to 80% confluence, mixed miRNA mimics (RiboBio, Guangzhou, China) at a concentration of 50 nM or inhibitors of miR-17-92 cluster components of 100 nM were transfected by using Lipofectamine 2000 (Invitrogen Corp, CA, USA) according to the manufacturers’ recommendations. Cells were then harvested for RNA extraction using TRIzol reagent at 48 h.

### Protein extraction and Western blot analyses

Total proteins were extracted and quantified using the BCA protein assay kit (Beyotime, China). For Western-blot analysis, equal amounts of total protein were loaded onto 8% SDS–PAGE gel, and subsequently electrotransferred to a nitrocellulose membrane. After blocking with 5% defatted milk in PBS-Tween-20 for 1 h at room temperature, the membranes were incubated with specific primary antibody overnight at 4 °C. After TBST washing, they were incubated with the appropriate secondary antibody at a 1:1000 dilution for 1 h at room temperature. GAPDH was used as an internal control. The protein bands were visualized by Immobilon Western Chemiluminescent HRP Substrate (Merck Millipore), and images were captured using Quantity One software (Bio-Rad). Antibodies to SAMD4, SAMD3, RB1, EP300 and GAPDH were purchased from Abcam.

### Dual luciferase reporter assays

The 3′ -UTR of SAMD4, SAMD3, RB1 and EP300 containing the putative target site for miR-17-92 cluster were cloned into the pGL3 vector (Genscript). The plasmid was cotransfected with pRL-SV40 and miRNA mimics into HEK 293 T cells using Lipofectamine™ 2000 (Invitrogen). At 48 h post transfection, the luciferase activities were measured with the Dual-Luciferase Reporter Assay System (Promega). Relative luciferase activity was normalized with Renilla luciferase activity.

### Cell proliferation assays

HTR-8/SVneo cells were seeded in a 96-well plate at a density of 1 × 10^4^ cells per well. At 24 h and 48 h after transfection with mimics, inhibitors or negative control, cell proliferation was measured using the Cell Counting Kit-8 (CCK-8) (Dojindo, Kumamoto, Japan) according to the protocol. The absorbance at a wavelength of 450 nm was measured with TECAN infinite M200 Multimode microplate reader (Tecan, Mechelen, Belgium) using culture medium as a blank. The cell proliferation % = (A in treated group - A in sham group)/(A in control group - A in sham group) × 100%. The experiments were repeated thrice independently.

### 5-Ethynyl-2′-deoxyuridine (EdU) assays

Proliferation of HTR-8/SVneo cells was also determined using the EdU DNA Proliferation *in vitro* Detection kit (RiboBio, China) according to manufacturer instructions. HTR-8/SVneo cells were seeded in 96-well plates and transfected with mimics, inhibitors or negative control. After 48 h trasfection, the cells were stained with EdU and DAPI. Images and data were automatically obtained from KineticScan™ reader (KSR; Cellomics, Pittsburgh, USA). These experiments were carried out thrice independently.

### Apoptosis assays

Cell apoptosis was conducted using Annexin V-FITC Kit (BD Biopharmingen, NJ, USA) according to the manufacturer’s protocol. After transfection (48 h), cells were harvested with trypsin, washed twice with PBS, and resuspended in binding buffer. After incubated with Annexin V-FITC at room temperature for 15 min, the cells were analyzed immediately by FACS Calibur Flow Cytometer (BD Biasciences, NJ, USA).

### Cell cycle analysis

For the determination of the cell cycle distribution, cells were collected after transfection (48 h), washed with cold PBS, fixed in cold 70% ethanol overnight at −20 °C. The fixed cells stained with PI (Sigma, MO, USA) at room temperature for 30 min. After staining, cell cycle distribution was analyzed with FACS Calibur Flow Cytometer. The cell cycle fraction was determined using Modfit LT version 3.0 software.

### Statistical analysis

All statistical analyses were performed with Stata (Version 9.0, StataCorp LP, TX, USA), and presented with GraphPad Prism software. Results are presented as mean ± standard error of the mean (SEM). Independent sample t-test (two tailed) was used to compare data between two groups. ROC curve analysis was carried out to evaluate the diagnostic effects of macrosomia using serum miRNAs of miR-17-92 cluster. *P* < 0.05 was considered statistically significant.

## Additional Information

**How to cite this article**: Li, J. *et al.* The role, mechanism and potentially novel biomarker of microRNA-17-92 cluster in macrosomia. *Sci. Rep.* **5**, 17212; doi: 10.1038/srep17212 (2015).

## Supplementary Material

Supplementary Information

## References

[b1] Lu Y., Zhang J., Lu X., Xi W. & Li Z. Secular trends of macrosomia in southeast China, 1994–2005. BMC public health 11, 818, 10.1186/1471-2458-11-818 (2011). 22011362 PMC3206484

[b2] Li G. *et al.* Prevalence of macrosomia and its risk factors in china: a multicentre survey based on birth data involving 101,723 singleton term infants. Paediatr. Perinat. Epidemiol. 28, 345–350, 10.1111/ppe.12133 (2014). 24891149

[b3] Zhang X., Decker A., Platt R. W. & Kramer M. S. How big is too big ? The perinatal consequences of fetal macrosomia. Am. J. Obstet. Gynecol. 198, 517 e511–516, 10.1016/j.ajog.2007.12.005 (2008). 18455528

[b4] Boney C. M., Verma A., Tucker R. & Vohr B. R. Metabolic syndrome in childhood: association with birth weight, maternal obesity, and gestational diabetes mellitus. Pediatrics 115, e290–296, 10.1542/peds.2004-1808 (2005). 15741354

[b5] Wei J. N. *et al.* Low birth weight and high birth weight infants are both at an increased risk to have type 2 diabetes among schoolchildren in taiwan. Diabetes care 26, 343–348 (2003). 12547860 10.2337/diacare.26.2.343

[b6] Yu Z. B. *et al.* Birth weight and subsequent risk of obesity: a systematic review and meta-analysis. Obes. Rev. 12, 525–542, 10.1111/j.1467-789X.2011.00867.x (2011). 21438992

[b7] Rastogi S. *et al.* Evaluating the Impact of a Pragmatic Nutrition Awareness Program for Expectant Mothers upon Birth Weight of the Newborn. Evid. Based Complement Alternat. Med. 2011, 185672, 10.1093/ecam/neq034 (2011). 21607010 PMC3094838

[b8] Jansson T. & Powell T. L. Role of the placenta in fetal programming: underlying mechanisms and potential interventional approaches. Clin. Sci. (Lond) 113, 1–13, CS20060339 (2007). 17536998 10.1042/CS20060339

[b9] Salafia C. M. *et al.* Placental characteristics and birthweight. Paediatr. Perinat. Epidemiol. 22, 229–239, 10.1111/j.1365-3016.2008.00935.x (2008). 18426518

[b10] Taricco E., Radaelli T., Nobile de Santis M. S. & Cetin I. Foetal and placental weights in relation to maternal characteristics in gestational diabetes. Placenta 24, 343–347 (2003). 12657507 10.1053/plac.2002.0913

[b11] Jones C. J. & Fox H. Syncytial knots and intervillous bridges in the human placenta: an ultrastructural study. J. Anat. 124, 275–286 (1977). 591426 PMC1234832

[b12] Unek G., Ozmen A., Mendilcioglu I., Simsek M. & Korgun E. T. Immunohistochemical distribution of cell cycle proteins p27, p57, cyclin D3, PCNA and Ki67 in normal and diabetic human placentas. J. Mol. Histol. 45, 21–34, 10.1007/s10735-013-9534-3 (2014). 23963898

[b13] Ujihira T. *et al.* MicroRNA-574-3p, identified by microRNA library-based functional screening, modulates tamoxifen response in breast cancer. Sci. Rep. 5, 7641, 10.1038/srep07641 (2015). 25560734 PMC4284514

[b14] Yu D. *et al.* Suppression of CYP2C9 by microRNA hsa-miR-128-3p in human liver cells and association with hepatocellular carcinoma. Sci. Rep. 5, 8534, 10.1038/srep08534 (2015). 25704921 PMC4336941

[b15] Mayor-Lynn K., Toloubeydokhti T., Cruz A. C. & Chegini N. Expression profile of microRNAs and mRNAs in human placentas from pregnancies complicated by preeclampsia and preterm labor. Reprod. Sci. 18, 46–56, 10.1177/1933719110374115 (2011). 21079238 PMC3343068

[b16] Lalevee S., Lapaire O. & Buhler M. miR455 is linked to hypoxia signaling and is deregulated in preeclampsia. Cell Death Dis. 5, e1408, 10.1038/cddis.2014.368 (2014). 25188518 PMC4540200

[b17] Doridot L. *et al.* miR-34a expression, epigenetic regulation, and function in human placental diseases. Epigenetics 9, 142–151, 10.4161/epi.26196 (2014). 24081307 PMC3928177

[b18] Yao Y. *et al.* Ancestral exposure to stress epigenetically programs preterm birth risk and adverse maternal and newborn outcomes. BMC Med. 12, 121, 10.1186/s12916-014-0121-6 (2014). 25286408 PMC4244860

[b19] Jiang H. *et al.* Aberrant upregulation of miR-21 in placental tissues of macrosomia. J. Perinatol. 34, 658–663, 10.1038/jp.2014.58 (2014). 24786382

[b20] Song Y. *et al.* MiR-18a regulates the proliferation, migration and invasion of human glioblastoma cell by targeting neogenin. Exp. Cell Res. 324, 54–64, 10.1016/j.yexcr.2014.03.009 (2014). 24657544

[b21] Chen D. B. & Wang W. Human placental microRNAs and preeclampsia. Biol. Reprod. 88, 130, 10.1095/biolreprod.113.107805 (2013). 23575145 PMC4013914

[b22] Ventura W. *et al.* Placental expression of microRNA-17 and -19b is down-regulated in early pregnancy loss. Eur. J. Obstet. Gynecol. Reprod. Biol. 169, 28–32, 10.1016/j.ejogrb.2013.01.025 (2013). 23433743

[b23] Mouillet J. F., Chu T. & Sadovsky Y. Expression patterns of placental microRNAs. Birth Defects Res. A Clin. Mol. Teratol. 91, 737–743, 10.1002/bdra.20782 (2011). 21425434 PMC5030720

[b24] Enquobahrie D. A. *et al.* Placental microRNA expression in pregnancies complicated by preeclampsia. Am. J. Obstet. Gynecol. 204, 178 e112–121, 10.1016/j.ajog.2010.09.004 (2011). PMC3040986 21093846

[b25] Ventura A. *et al.* Targeted deletion reveals essential and overlapping functions of the miR-17 through 92 family of miRNA clusters. Cell 132, 875–886, 10.1016/j.cell.2008.02.019 (2008). 18329372 PMC2323338

[b26] Wang W. *et al.* Preeclampsia up-regulates angiogenesis-associated microRNA (i.e., miR-17, -20a, and -20b) that target ephrin-B2 and EPHB4 in human placenta. J. Clin. Endocrinol. Metab. 97, E1051–1059, 10.1210/jc.2011-3131 (2012). 22438230 PMC3387422

[b27] Li J. *et al.* A MicroRNA Signature in Gestational Diabetes Mellitus Associated with Risk of Macrosomia. Cell. Physiol. Biochem. 37, 243–252, 10.1159/000430349 (2015). 26302821

[b28] Cho W. C. OncomiRs: the discovery and progress of microRNAs in cancers. Mol. Cancer 6, 60, 10.1186/1476-4598-6-60 (2007). 17894887 PMC2098778

[b29] Olive V., Jiang I. & He L. mir-17-92, a cluster of miRNAs in the midst of the cancer network. Int. J. Biochem. Cell Biol. 42, 1348–1354, 10.1016/j.biocel.2010.03.004 (2010). 20227518 PMC3681296

[b30] Soundararajan R. & Rao A. J. Trophoblast ‘pseudo-tumorigenesis’: significance and contributory factors. Reprod. Biol. Endocrinol. 2, 15, 10.1186/1477-7827-2-15 (2004). 15043753 PMC407853

[b31] Kumar P., Luo Y., Tudela C., Alexander J. M. & Mendelson C. R. The c-Myc-regulated microRNA-17~92 (miR-17~92) and miR-106a~363 clusters target hCYP19A1 and hGCM1 to inhibit human trophoblast differentiation. Mol. Cell. Biol. 33, 1782–1796, 10.1128/MCB.01228-12 (2013). 23438603 PMC3624183

[b32] Bueno M. J. & Malumbres M. MicroRNAs and the cell cycle. Biochim. Biophys. Acta 1812, 592–601, 10.1016/j.bbadis.2011.02.002 (2011). 21315819

[b33] Massague J. TGF-beta signal transduction. Annu. Rev. Biochem. 67, 753–791, 10.1146/annurev.biochem.67.1.753 (1998). 9759503

[b34] Mestdagh P. *et al.* The miR-17-92 microRNA cluster regulates multiple components of the TGF-beta pathway in neuroblastoma. Mol. Cell 40, 762–773, 10.1016/j.molcel.2010.11.038 (2010). 21145484 PMC3032380

[b35] Dews M. *et al.* The myc-miR-17~92 axis blunts TGF{beta} signaling and production of multiple TGF{beta}-dependent antiangiogenic factors. Cancer Res. 70, 8233–8246, 10.1158/0008-5472.CAN-10-2412 (2010). 20940405 PMC3007123

[b36] Xuan Y. H. *et al.* Expression of TGF-beta signaling proteins in normal placenta and gestational trophoblastic disease. Histol. Histopathol. 22, 227–234 (2007). 17163397 10.14670/HH-22.227

[b37] Li Y. *et al.* The impact of TGF-beta1 on the mRNA expression of TbetaR I, TbetaR II, Smad4 and the invasiveness of the JEG-3 placental choriocarcinoma cell line. Oncol. Lett. 4, 1344–1348, 10.3892/ol.2012.906 (2012). 23205135 PMC3506760

[b38] Wells D. *et al.* Expression of genes regulating chromosome segregation, the cell cycle and apoptosis during human preimplantation development. Hum. Reprod. 20, 1339–1348, 10.1093/humrep/deh778 (2005). 15705620

[b39] Wu L. *et al.* Extra-embryonic function of Rb is essential for embryonic development and viability. Nature 421, 942–947, 10.1038/nature01417 (2003). 12607001

[b40] Rajaraman G., Murthi P., Pathirage N., Brennecke S. P. & Kalionis B. Downstream targets of homeobox gene HLX show altered expression in human idiopathic fetal growth restriction. Am. J. Pathol. 176, 278–287, 10.2353/ajpath.2010.090187 (2010). 20008130 PMC2797890

[b41] Miura K. *et al.* Identification of pregnancy-associated microRNAs in maternal plasma. Clin. Chem. 56, 1767–1771, 10.1373/clinchem.2010.147660 (2010). 20729298

[b42] Olson E. N. MicroRNAs as therapeutic targets and biomarkers of cardiovascular disease. Sci. Transl. Med. 6, 239ps233, 10.1126/scitranslmed.3009008 (2014). PMC4279862 24898744

[b43] Hu L. *et al.* Early second-trimester serum microRNAs as potential biomarker for nondiabetic macrosomia. Biomed. Res. Int. 2014, 394125, 10.1155/2014/394125 (2014). 25405200 PMC4227359

[b44] Jiang H. *et al.* Serum MicroRNAs as Diagnostic Biomarkers for Macrosomia. Reprod. Sci. 22, 664–671, 10.1177/1933719114561557 (2015). 25519717 PMC4502808

[b45] Ge Q. *et al.* Differential expression of circulating miRNAs in maternal plasma in pregnancies with fetal macrosomia. Int. J. Mol. Med. 35, 81–91, 10.3892/ijmm.2014.1989 (2015). 25370776 PMC4249743

[b46] Mouillet J. F. *et al.* The levels of hypoxia-regulated microRNAs in plasma of pregnant women with fetal growth restriction. Placenta 31, 781–784, 10.1016/j.placenta.2010.07.001 (2010). 20667590 PMC3204658

[b47] Mincheva-Nilsson L. & Baranov V. The role of placental exosomes in reproduction. Am. J. Reprod. Immunol. 63, 520–533, 10.1111/j.1600-0897.2010.00822.x (2010). 20331583

[b48] Luo S. S. *et al.* Human villous trophoblasts express and secrete placenta-specific microRNAs into maternal circulation via exosomes. Biol. Reprod. 81, 717–729, 10.1095/biolreprod.108.075481 (2009). 19494253

[b49] Hsu S. D. *et al.* miRTarBase: a database curates experimentally validated microRNA-target interactions. Nucleic Acids Res. 39, D163–169, 10.1093/nar/gkq1107 (2011). 21071411 PMC3013699

