# Supplementary material for: The role, mechanism and potentially novel biomarker of microRNA-17-92 cluster in macrosomia
Source: Sci Rep. 2015 Nov 24;5:17212. doi: 10.1038/srep17212 (PMC4657041; doi:10.1038/srep17212)
Supplement: Supplementary Information [file srep17212-s1.doc]

**The role, mechanism and potentially novel biomarker of microRNA-17-92 cluster in macrosomia**

Jing Li Ph.D., Liping Chen Ph.D., Qiuqin Tang M.S., Wei Wu Ph.D., Hao Gu M.S., Lou Liu M.S., Jie Wu M.S., Hua Jiang M.S., Hongjuan Ding M.D., Yankai Xia Ph.D., Daozhen Chen Ph.D., Yali Hu Ph.D., Xinru Wang Ph.D.

**Additional file 1: Table S1.** Clinical characteristics of the study population.

| **Characteristic** | **Control (n = 100)** | **Macrosomia (n = 57)** | ***P* value** |
| --- | --- | --- | --- |
| **Maternal age (years)** | 28.30 ± 4.23 | 27.48 ± 2.90 | NS |
| **Gestational age (weeks)** | 38.86 ± 3.95 | 39.53 ± 0.85 | NS |
| **Maternal BMI before pregnancy ( kg/m^2^)** | 24.07 ± 4.80 | 22.40 ± 3.94 | < 0.05 |
| **Weight gain during pregnancy (kg)** | 18.12 ± 3.93 | 19.28 ± 3.89 | NS |
| **Birth weight (g)** | 3438.2 ± 317.18 | 4194.56 ± 166.23 | < 0.01 |
| **Infant gender, n (%)** |  |  | NS |
| **Male** | 51 (51) | 37 (64.91) |  |
| **Female** | 49 (49) | 20 (35.09) |  |

Values are mean ± SD. BMI, body mass index. NS = not significant.

**Additional file 2: Table S2.** Detailed information of miR-17-92 target genes in cell cycle pathway.

| **Gene** | **Official full name** | **Location** | **miRNA** |
| --- | --- | --- | --- |
| CCNL1 | cyclin L1 | 3q25.31 | 18a-5p, 19b-3p |
| SMAD4 | SMAD family member 4 | 18q21.1 | 18a-5p, 19a-3p, 19b-3p, 20a-5p, 92a-3p |
| ATM | ATM serine/threonine kinase | 11q22-q23 | 18a-5p, 92a-3p |
| SMAD3 | SMAD family member 3 | 15q22.33 | 18a-5p |
| MCM5 | minichromosome maintenance complex component 5 | 22q13.1 | 18a-5p |
| CDC20 | cell division cycle 20 | 1p34.1 | 18a-5p, 92a-3p |
| PCNA | proliferating cell nuclear antigen | 20pter-p12 | 18a-5p |
| PKMYT1 | protein kinase, membrane associated tyrosine/threonine 1 | 16p13.3 | 18a-5p |
| CCND1 | cyclin D1 | 11q13 | 19b-3p, 20a-5p, 92a-3p |
| WEE1 | WEE1 G2 checkpoint kinase | 11p15.4 | 19b-3p, 20a-5p, 92a-3p |
| CCND2 | cyclin D2 | 12p13 | 19b-3p, 20a-5p |
| E2F1 | E2F transcription factor 1 | 20q11.2 | 20a-5p |
| CDKN1A | cyclin-dependent kinase inhibitor 1A (p21, Cip1) | 6p21.2 | 20a-5p |
| E2F3 | E2F transcription factor 3 | 6p22 | 20a-5p |
| RB1 | retinoblastoma 1 | 13q14.2 | 20a-5p |
| RBL1 | retinoblastoma-like 1 | 20q11.2 | 20a-5p |
| RBL2 | retinoblastoma-like 2 | 16q12.2 | 20a-5p |
| MAD1L1 | MAD1 mitotic arrest deficient-like 1 (yeast) | 7p22 | 20a-5p |
| CCNB1 | cyclin B1 | 5q12 | 20a-5p, 92a-3p |
| CDH1 | cadherin 1, type 1, E-cadherin (epithelial) | 16q22.1 | 92a-3p |
| CDK1 | cyclin-dependent kinase 1 | 10q21.1 | 92a-3p |
| HDAC1 | histone deacetylase 1 [Homo sapiens | 1p34 | 92a-3p |
| CDC25A | cell division cycle 25A | 3p21 | 92a-3p |
| MCM3 | minichromosome maintenance complex component 3 | 6p12 | 92a-3p |
| RBX1 | ring-box 1, E3 ubiquitin protein ligase | 22q13.2 | 92a-3p |
| YWHAQ | tyrosine 3-monooxygenase/tryptophan 5-monooxygenase activation protein,theta | 2p25.1 | 92a-3p |
| MDM2 | MDM2 proto-oncogene, E3 ubiquitin protein ligase | 12q14.3-q15 | 92a-3p |
| GSK3B | glycogen synthase kinase 3 beta | 3q13.3 | 92a-3p |
| ORC5 | origin recognition complex, subunit 5 | 7q22.1 | 92a-3p |
| CCNE1 | cyclin E1 | 19q12 | 92a-3p |
| SMC1A | structural maintenance of chromosomes 1A | Xp11.22-p11.21 | 92a-3p |
| ORC6 | origin recognition complex, subunit 6 | 16q12 | 92a-3p |
| EP300 | E1A binding protein p300 | 22q13.2 | 92a-3p |
| CHEK1 | checkpoint kinase 1 | 11q24.2 | 92a-3p |
| MCM7 | minichromosome maintenance complex component 7 | 7q21.3-q22.1 | 92a-3p |

**Additional file 3: Figure S1. Expression levels of miR-17-92 cluster miRNAs in HTR8/SVneo cells transfected with miR-17-92 mimics or inhibitors.** Data are expressed as the mean ± SEM (n = 3). * *P* < 0.05; ** *P* < 0.01; compared with the control group.


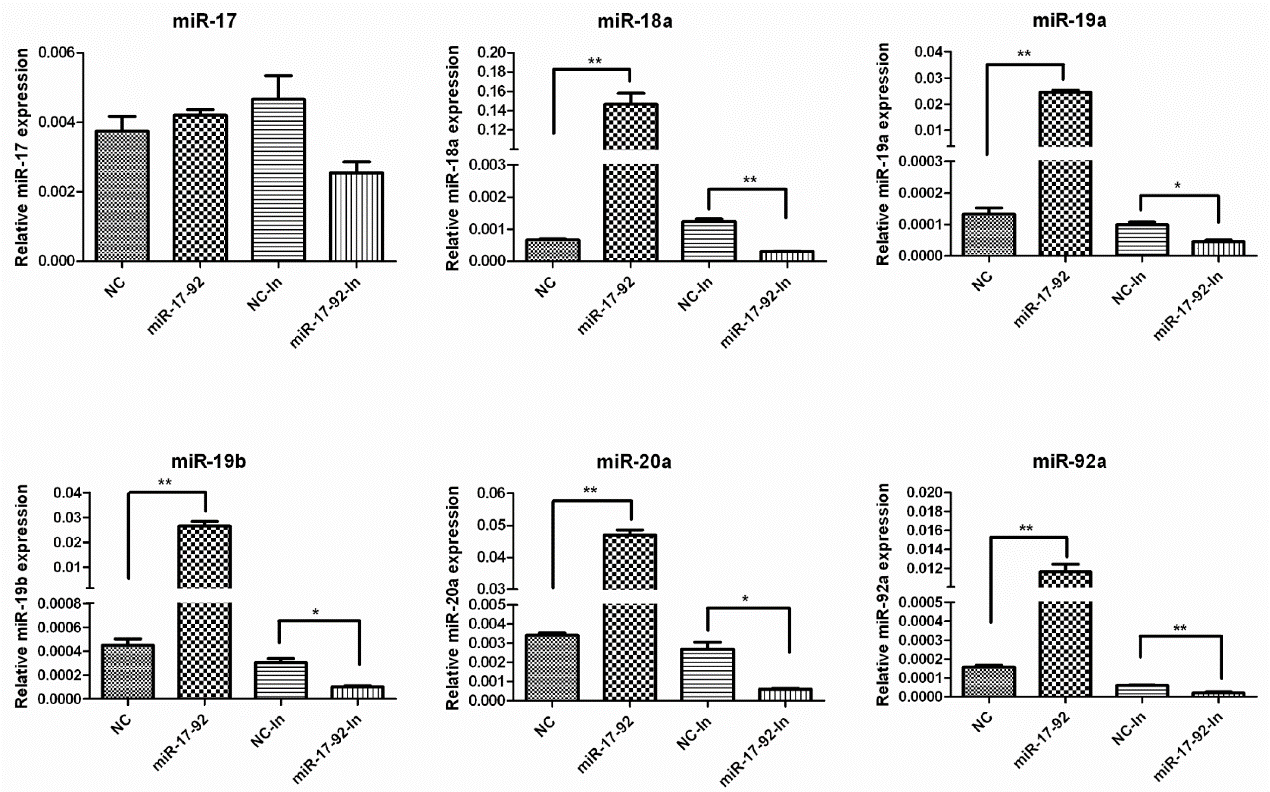


**Additional file 4: Figure S2. Expression levels of 35 target genes of miR-17-92 cluster miRNAs in cell cycle pathway.** Data are expressed as the mean ± SEM (n = 3). * *P* < 0.05; ** *P* < 0.01; compared with the control group.


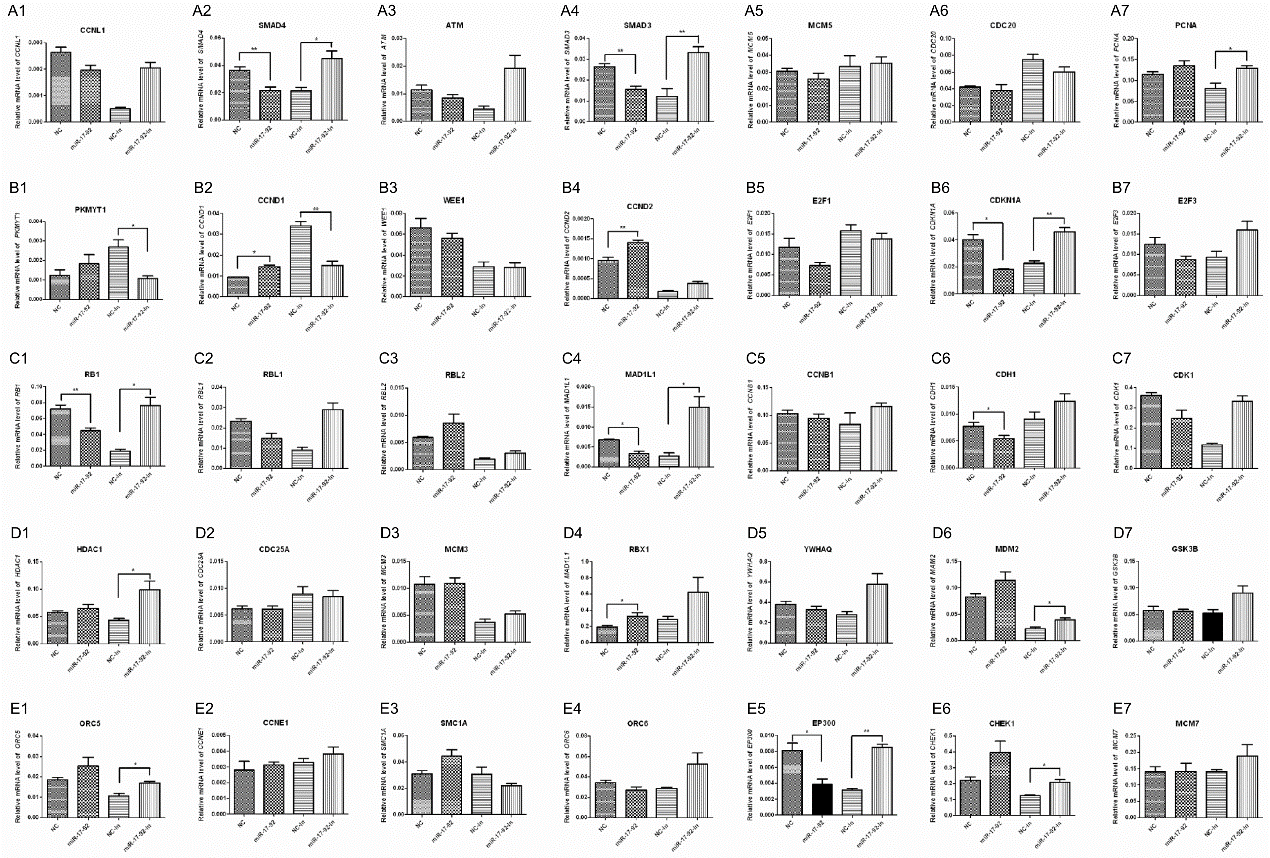


**Additional file 5: Figure S3. miR-17-92 cluster targeted multiple negative regulators of cell cycle pathway.** (A) Detection of *SMAD4*, *SMAD3*, *CDKNIA*, *RB1*, *MAD1L1* and *EP300* mRNA expression in placentas of macrosomia (n = 57) and those with a normal controls (n = 100) were assessed with qRT-PCR, and normalized to *GAPDH*. (B) Protein expression of SMAD4, SMAD3, RB1, and EP300 were analyzed by Western-blot in placentas of macrosomia (n = 3) and those with a normal controls (n = 3). Data were normalized to the level of GAPDH. * *P* < 0.05; ** *P* < 0.01; compared with the control group.

**
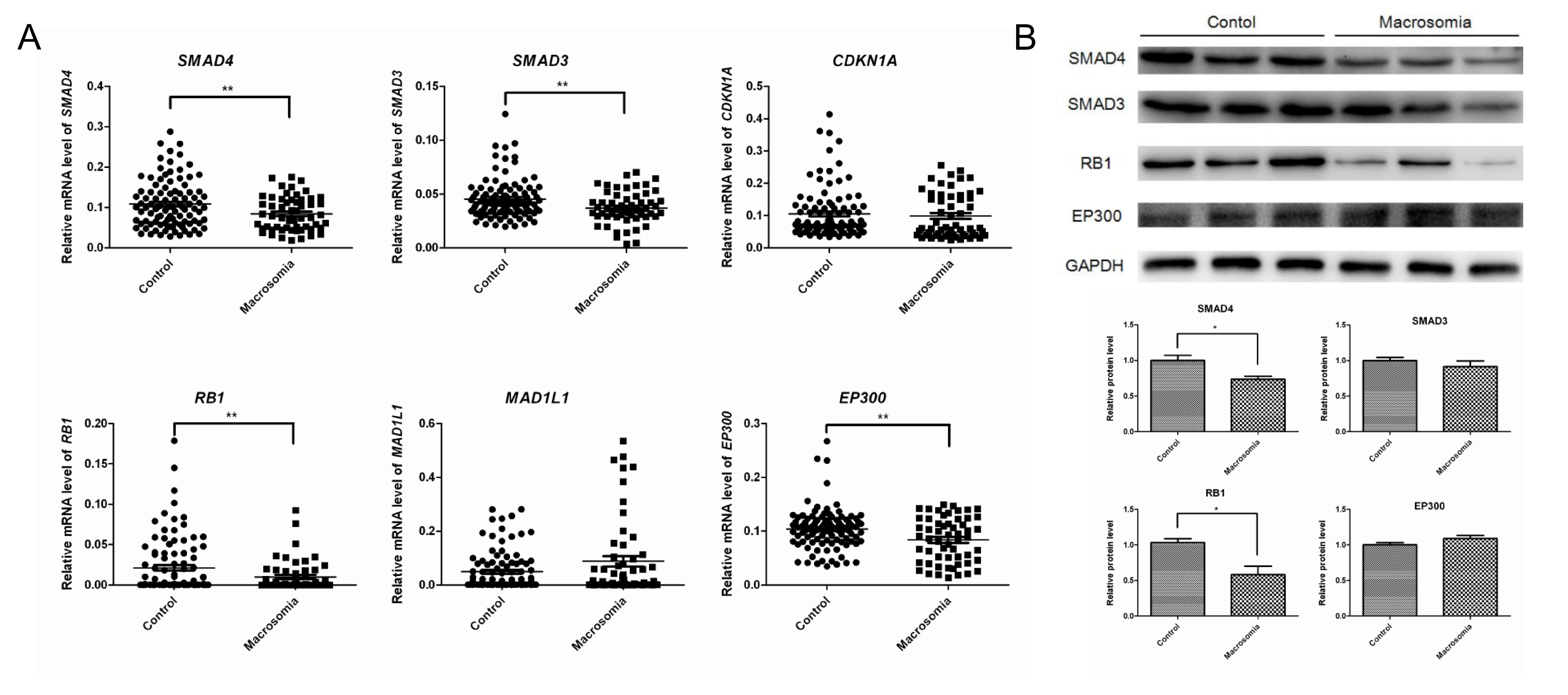
**

**Additional file 6: Figure S4. *DROSHA* and *DICER* were overexpressed in placentas of macrosomia.** Expression levels of *DROSHA* (A) and *DICER* (B) were analyzed by qRT-PCR, and normalized to *GAPDH* in macrosomia (n = 57) and controls (n = 100). ** *P* < 0.01; compared with the control group.


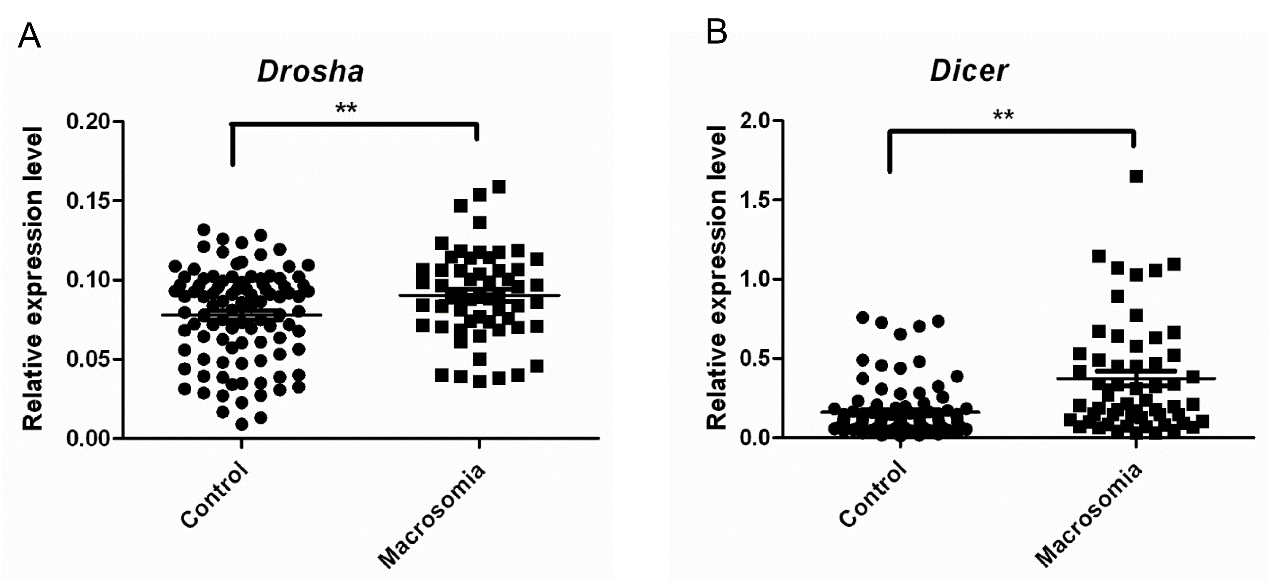


**Additional file 7: Figure S5. Expression levels of miR-17-92 cluster in maternal serum of macrosomia and controls.** Expression levels of miR-17-92 cluster in maternal serum of macrosomia (n = 23) and controls (n = 23) were determined by qRT-PCR, and normalized to RNU6B. * *P* < 0.05; compared with the control group.


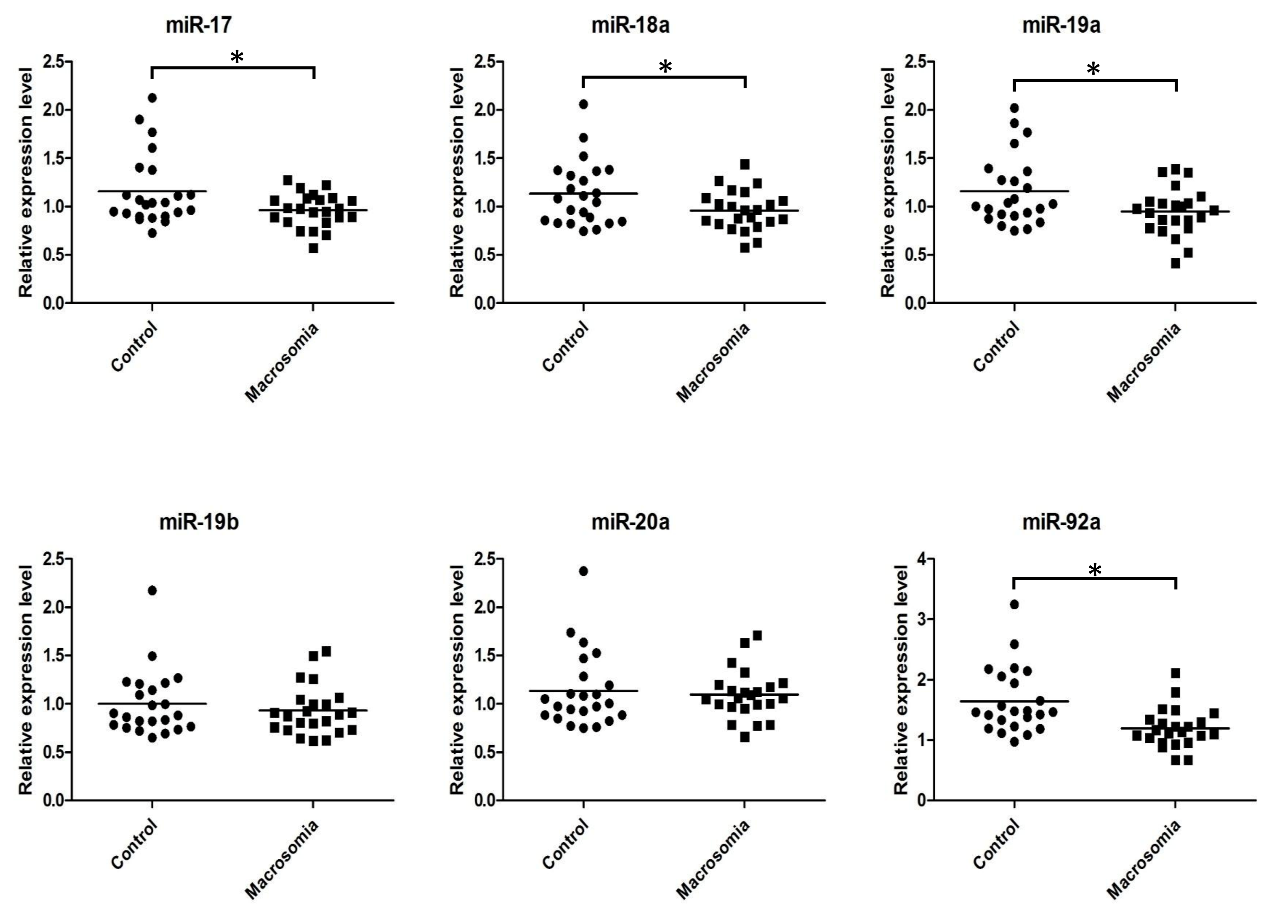


**Additional file 8: Table S3.** Sequences of primers for qRT-PCR analyses of miR-17-92 miRNAs.

| **miRNA name** | **Forward primer** | **Reverse primer** |
| --- | --- | --- |
| hsa-miR-17-5p | ACACTCCAGCTGGGCAAAGTGCTTACAGTGC | CTCAACTGGTGTCGTGGAGTCGGCAATTCAGTTGAGCTACCTGC |
| hsa-miR-18a-5p | ACACTCCAGCTGGGTAAGGTGCATCTAGTGC | CTCAACTGGTGTCGTGGAGTCGGCAATTCAGTTGAGCTATCTGC |
| hsa-miR-19a-3p | ACACTCCAGCTGGGTGTGCAAATCTATGCAA | CTCAACTGGTGTCGTGGAGTCGGCAATTCAGTTGAGTCAGTTTT |
| hsa-miR-19b-3p | ACACTCCAGCTGGGTGTGCAAATCCATGCAA | CTCAACTGGTGTCGTGGAGTCGGCAATTCAGTTGAGTCAGTTTT |
| hsa-miR-20a-5p | ACACTCCAGCTGGGTAAAGTGCTTATAGTGC | CTCAACTGGTGTCGTGGAGTCGGCAATTCAGTTGAGCTACCTGC |
| hsa-miR-92a-3p | ACACTCCAGCTGGGTATTGCACTTGTCCCG | CTCAACTGGTGTCGTGGAGTCGGCAATTCAGTTGAGACAGGCCG |
| U6 | CTCGCTTCGGCAGCACA | AACGCTTCACGAATTTGCGT |
| URP | TGGTGTCGTGGAGTCG |  |

**Additional file 9: Table S4.** Sequences of primers for qRT-PCR analyses of miR-17-92 target genes.

| **Gene name** | **Forward primer** | **Reverse primer** |
| --- | --- | --- |
| CCNL1 | TACCATCGACCACTCTCTGATT | GGATGCGTAAGTCCGTCTCAC |
| SMAD4 | CTCATGTGATCTATGCCCGTC | AGGTGATACAACTCGTTCGTAGT |
| ATM | ATCTGCTGCCGTCAACTAGAA | GATCTCGAATCAGGCGCTTAAA |
| SMAD3 | CCATCTCCTACTACGAGCTGAA | CACTGCTGCATTCCTGTTGAC |
| MCM5 | AGCATTCGTAGCCTGAAGTCG | CGGCACTGGATAGAGATGCG |
| CDC20 | GACCACTCCTAGCAAACCTGG | GGGCGTCTGGCTGTTTTCA |
| PCNA | CCTGCTGGGATATTAGCTCCA | CAGCGGTAGGTGTCGAAGC |
| PKMYT1 | CATGGCTCCTACGGAGAGGT | ACATGGAACGCTTTACCGCAT |
| CCND1 | GCTGCGAAGTGGAAACCATC | CCTCCTTCTGCACACATTTGAA |
| WEE1 | AGGGAATTTGATGTGCGACAG | CTTCAAGCTCATAATCACTGGCT |
| CCND2 | ACCTTCCGCAGTGCTCCTA | CCCAGCCAAGAAACGGTCC |
| E2F1 | ACGTGACGTGTCAGGACCT | GATCGGGCCTTGTTTGCTCTT |
| CDKN1A | TGTCCGTCAGAACCCATGC | AAAGTCGAAGTTCCATCGCTC |
| E2F3 | AGAAAGCGGTCATCAGTACCT | TGGACTTCGTAGTGCAGCTCT |
| RB1 | CTCTCGTCAGGCTTGAGTTTG | GACATCTCATCTAGGTCAACTGC |
| RBL1 | CTGGACGACTTTACTGCCATC | TCCAACCGTGGGAATAATGCT |
| RBL2 | CCACCCCTCAGATCCAGCA | CGTGTAGCTTTCGCTCATGC |
| MAD1L1 | TGGACTGGATATTTCTACCTCGG | CCTCACGCTCGTAGTTCCTG |
| CCNB1 | AATAAGGCGAAGATCAACATGGC | TTTGTTACCAATGTCCCCAAGAG |
| CDH1 | AGCCAACTGGAGCGTGAAC | TCTTTGCCGTTGTCTGAGGTG |
| CDK1 | AAACTACAGGTCAAGTGGTAGCC | TCCTGCATAAGCACATCCTGA |
| HDAC1 | CTACTACGACGGGGATGTTGG | GAGTCATGCGGATTCGGTGAG |
| CDC25A | GTGAAGGCGCTATTTGGCG | TGGTTGCTCATAATCACTGCC |
| MCM3 | TCAGAGAGATTACCTGGACTTCC | TCAGCCGGTATTGGTTGTCAC |
| RBX1 | TTGTGGTTGATAACTGTGCCAT | GACGCCTGGTTAGCTTGACAT |
| YWHAQ | AGGGTCATCTCTAGCATCGAG | CCACTTTCTCCCGATAGTCCTT |
| MDM2 | CAGTAGCAGTGAATCTACAGGGA | CTGATCCAACCAATCACCTGAAT |
| GSK3B | AGACGCTCCCTGTGATTTATGT | CCGATGGCAGATTCCAAAGG |
| ORC5 | GAAAACGTGGTGCTTTGTCG | GGTCTTTCCACTAGCAGTATGTC |
| CCNE1 | AAGGAGCGGGACACCATGA | ACGGTCACGTTTGCCTTCC |
| SMC1A | AACCTGCGGGTAAAGACCCT | GGCAAAGGTACGGTCCTCAG |
| ORC6 | ACAAGGAGACATATCAGAGCTGT | AGTGGCCTGGATAAGTCAAGAT |
| EP300 | GCTTCAGACAAGTCTTGGCAT | ACTACCAGATCGCAGCAATTC |
| CHEK1 | ATATGAAGCGTGCCGTAGACT | TGCCTATGTCTGGCTCTATTCTG |
| MCM7 | CCTACCAGCCGATCCAGTCT | CCTCCTGAGCGGTTGGTTT |
| DICER | GAGCTGTCCTATCAGATCAGGG | ACTTGTTGAGCAACCTGGTTT |
| DROSHA | CATGTCACAGAATGTCGTTCCA | GGGTGAAGCAGCCTCAGATTT |
| GAPDH | GCACCGTCAAGGCTGAGAAC | GGATCTCGCTCCTGGAAGATG |
